# Hypovitaminosis D and Mild Hypocalcaemia Are Highly Prevalent among Young Vietnamese Children and Women and Related to Low Dietary Intake

**DOI:** 10.1371/journal.pone.0063979

**Published:** 2013-05-24

**Authors:** Arnaud Laillou, Frank Wieringa, Thuy Nga Tran, Pham Thuy Van, Bach Mai Le, Sonia Fortin, Thi Hop Le, Regina Moench Pfanner, Jacques Berger

**Affiliations:** 1 Global Alliance for Improved Nutrition (GAIN), Geneva, Switzerland; 2 National Institute of Nutrition (NIN), Hanoi, Vietnam; 3 Institut de Recherche pour le Développement (IRD), UMR Nutripass IRD-UM2-UM1, Montpellier, France; Charité University Medicine Berlin, Germany

## Abstract

**Introduction:**

In many developing countries including Vietnam, data are lacking on vitamin D and calcium deficiencies whereas those deficiencies can play an important role in the development of bone health and possibly non-communicable diseases. The purpose of this study was to determine the overall prevalence of vitamin D and calcium deficiencies in women and young children and their nutritional related risk factors.

**Methods:**

A cross-sectional study conducted among 595 women of reproductive age and 532 children <5 years from 19 provinces of Vietnam. For each individual, data concerning daily diet, socioeconomic group, anthropometric status were obtained, and plasma concentrations of calcium and vitamin D were measured.

**Results:**

The prevalence of hypovitaminosis D status was very high, with the prevalence of vitamin D deficiency (25(OH)D<30 nmol/L) and insufficiency (25(OH)D between 30–49.9 nmol/L) being 17% and 40% in women and 21% and 37% in children, respectively. Using more liberal cut-off of 75 nmol/L, approximately 90% of the women and children were classified as having hypovitaminosis D. Overweight/obese women had a 2 times lower risk (OR = 0.46, [0.24–0.90]) for vitamin D deficiency than non-overweight and non-obese women. No participant had severe calcium deficiency but moderate and mild hypocalcaemia (plasma calcium concentrations between 1.15-0.9 mmol/L for mild deficiency and between 0.9-0.8 mmol/L for moderate deficiency) affected respectively 14% and 83% of the women with 97% of the children having mild hypocalcaemia. Women and children consumed about 1% of the Institute of Medicine (IOM) recommended nutrient intake (RNI) for vitamin D and less than 43% of the RNI for calcium.

**Conclusion:**

Our study suggests that calcium and vitamin D deficiencies represent a major public health concern in Vietnam. Thus, actions to improve the vitamin D and calcium status of the Vietnamese population should be considered.

## Introduction

Calcium and vitamin D are essential nutrients for the human body throughout the life and are closely linked as vitamin D deficiency can reduce intestinal absorption of calcium [1] and low dietary intake of calcium results in increased catabolism of vitamin D [2], [3]. In children, Vitamin D deficiency can cause growth retardation and rickets [4]. Low dietary calcium and vitamin D intake, low exposure to sunlight and high phytate intake combine to induce vitamin D deficiency and rickets [4]. Studies among Asian children and African American teenagers also suggested that low dietary intake of calcium induce the development of Vitamin D deficiency and rickets [2]. In adults, hypovitaminosis D will exacerbate both osteopenia and osteoporosis and increase the risk of fracture [3]–[5]. In addition, several studies have suggested that vitamin D supplementation is involved in reducing cardiovascular diseases and risk [6]. A prospective study conducted among 18,225 men assessed the association between serum 25(OH)D and risk of coronary disease, those with serum 25(OH)D ≤37 nmol/L) having higher risk of developing myocardial infarction, compared with those with levels of vitamin D ≥75 nmol/L) [7]. Another study followed 3,000 Caucasian patients who were routinely referred to coronary angiography for seven years and found that patients with severe hypovitaminosis D, defined as 25(OH)D <25 nmol/L, had a risk of dying from heart failure or sudden cardiac death that was three to five times greater than among patients with optimal levels of vitamin D [8]. Vitamin D may offer preventive and therapeutic benefits but the role of vitamin D is complex and requires further trials to define whether non-skeletal effects can be directly attributed to the increase of vitamin D intake [9].

Reported prevalence of vitamin D deficiency and insufficiency depend on the cut-off values used that vary between studies. An estimated 1 billion people worldwide have vitamin D deficiency or insufficiency [3]. Increasingly, hypovitaminosis D is being reported in controlled trials from Asia and therefore is not nationally representative. In Pakistan, the prevalence of hypovitaminosis D (25(OH)D <37.5 nmol/L) is reported in 66.7% infants and 81.1% of breastfeeding mothers [10]. In India, 48% of mothers and 52% of infants have 25(OH)D less than 25 nmol/L [11]. In China, 89% of adolescent girls in Beijing have serum 25(OH)D <50 nmol/L [12]. Hypovitaminosis D, defined by a 25(OH)D concentration <50 nmol/L, has also been shown in 90% of women from Hong Kong [13] and 60% Indonesian women [14], and in 72% of primary school children in Malaysia [15], despite living close to the latitude of equator.

Evidence suggesting a high prevalence of calcium deficiency exists in both developed and developing countries. For example, in the United Kingdom, it is estimated that 13% to 18% of women between the ages of 14 to 34 years and 8% to 15% of those over 65 years are calcium deficient [16]. Regarding the developing world, habitual calcium intake is estimated at only 25% to 33% of the adequate intake for many populations and age groups [17].

In Vietnam, data on vitamin D and calcium are sparse or carried out on limited samples of populations [18], [19]. As a result, the Vietnamese Ministry of Health requested a study in order to obtain information on these deficiencies nationwide. The main objective of the present study was thus to determine the prevalence of vitamin D and calcium deficiencies in women and young children included in a micronutrient survey carried out in 19 provinces of Vietnam at the end of 2010 and their nutritional-related risk factors.

## Materials and Methods

### Study Design and Sampling

In 2009, a nationwide food consumption survey was carried in Vietnam among young children and women of reproductive age from 7,680 households randomly selected using a stratified 2-stage cluster sampling procedure with probability proportionate to size (104 urban and 408 rural clusters). In 2010, the same participants were re-surveyed to provide additional information on micronutrient status of women in reproductive age and young children. The 2010 micronutrient study [20] was carried-out in a subset of 840 same households from 56 urban and 56 rural clusters (15 households per cluster) from 19 randomly selected provinces, 6 from the North, 5 from the Center and 8 from the South showing a geographically balanced distribution.

**Figure 1:**
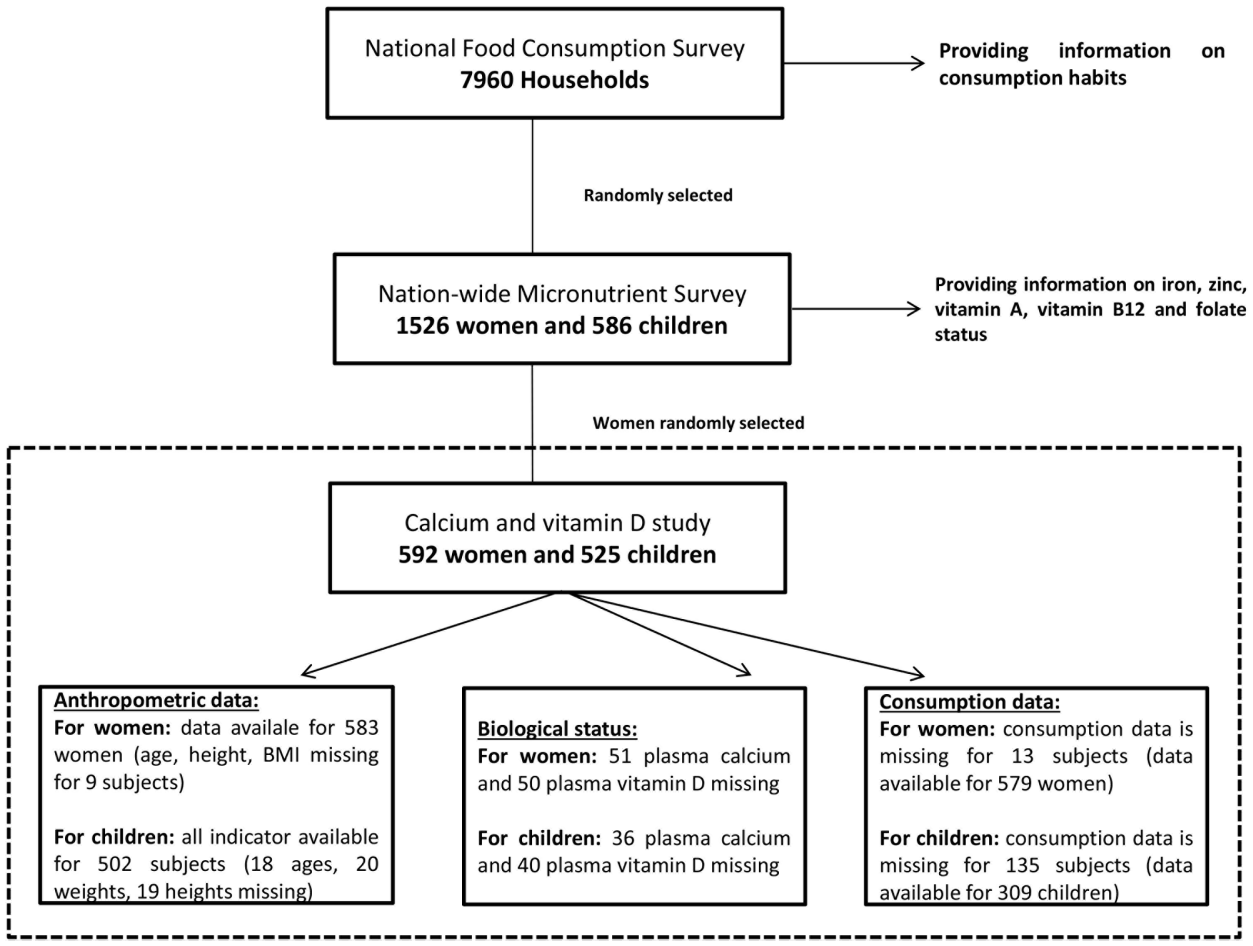
Study profile. Note: The population analyzed for this article is a subsample of the National Food Consumption Survey sample that was also used for the Nationwide Vitamin and Mineral Deficiencies Study. However, this subsample has not biased the results, as similar findings on dietary calcium intake were found among the 7980 households surveyed [53].

For calcium and vitamin D, all the children randomly selected from the 2010 study were included [20]. For the women of reproductive age, the sample size needed was estimated to be 267 subjects to obtain information on the entire population. With an expected design effect of 2.0, a minimum sample size of 538 subjects was calculated to get a precision of 5.0%. To allow for potential absence of a plasma sample 592 women were randomly selected from the list of the 1,520 women selected for the other micronutrients [20], stratified for province and rural/urban living area. Selected plasma samples were analyzed for calcium and vitamin D concentrations. The study profile is presented in figure 1.

### Anthropometry

Selected women and children were invited to the Commune Health Center early morning. Children and women were weighed without shoes or sandals and wearing light clothes by using a balance with a precision of 0.1 kg (Body Composition Monitor Scale Tanita BC-543, Japan). Height was measured by using a height measuring device with a precision of 0.1 cm (wooden height board, UNICEF). For children under two years, measurement of recumbent length was taken on an adjustable child length measuring board with a precision of 0.1 cm (wooden length board, UNICEF).

For children from 6 months to 6 years, anthropometric z-scores were calculated based on the National Center for Health Statistics/WHO growth reference data of 2006 [21], and using the Access Visual Basic program incorporating tables from the WHO Anthro software for standard population. Anthropometric status was assessed by the following indicators: weight-for-age<-2z-scores (WAZ) for underweight, height-for-age<-2 z-scores (HAZ) for stunting and weight-for-height<-2 z-scores (WHZ) for wasting. Overweight/obesity is defined as a Body Mass Index-z-scores for age (BMI-z)>2 z-scores [22].

For women, the body mass index (BMI) was calculated as the body weight (in kg) divided by the square of height (in meters). Women were classified using the BMI cut-off points endorsed for Asian populations: underweight (BMI <18.5 kg/m2), healthy weight (BMI 18.5–22.9 kg/m2), overweight (BMI 23.0–27.49 kg/m2), and obese (BMI ≥27.5 kg/m2) [23].

### Blood Sampling and Analysis

Just after anthropometry, a non-fasting blood sample was collected between 0700 and 0900 hours from women and children from the selected HH. Venous blood, 6 ml for women and 4 ml for children, was drawn by venipuncture in trace-element free heparinized sampling tubes (Vacuette, Greiner Bio One, Austria) by licensed medical technicians at the health center of the commune. Blood samples were stored in the dark in a cool box and transported within 4 hours of collection to the laboratory of Provincial Medical Center. Thereafter, plasma was obtained by centrifugation at 3000 g for 10 min at 4°C. Plasma was aliquoted into 200 µL pre-labeled Eppendorf tubes and kept frozen at −20°C at the PMC before being sent (within two weeks) on dry ice to the National Institute of Nutrition (NIN) where samples were stored at −70°C until analysis.

### Vitamin D and Calcium Measurements

The Micronutrient Laboratory of the National Institute of Nutrition (NIN) performed the analysis of plasma vitamin D. Vitamin D status was assessed by measuring the serum concentration of 25-hydroxyvitamin D. Serum/plasma 25(OH)D is considered the best indicator for vitamin D status, since it is not tightly regulated by parathyroid hormone and is not affected by dietary calcium and phosphorus intake [24].Serum 25-OH vitamin D (25(OH)D) concentration was determined by HPLC (Alliance, Waters, USA) according to the method described by Turpeinen [25]. The quality controls and standards of 25(OH)D were respectively from the National Institute of Standards and Technology (NIST, USA) and from Immundiagnostik AG, Germany to calibrate the equipment. Plasma ionized calcium was measured with an ion-sensitive electrode on a Roche Diagnostics AVL 9180 instrument (AVL 9180, Roche, Japan) at the Medicine Laboratory Technology Co., Ltd, Hanoi, Vietnam. The quality controls and standards for ionized calcium were purchased from Roche. The within-assay variability for plasma vitamin D and ionized calcium was <6%.

### Vitamin D and Calcium Status

In this study vitamin D deficiency (VDD) and insufficiency (VDI) were defined according to the Institute of Medicine (IOM) criteria [26]as a concentration of 25(OH)D below 30 nmol/L was considered as VDD, between 30–49.9 nmol/L indicated VDI and above 50 nmol/L a sufficient status. However, different cut-offs have been used, and two meta-analyses by Bischoff-Ferrari and colleagues demonstrated that serum vitamin D below concentrations of 75 nmol/L were associated with bone fractures, and for certain types of fractures even for vitamin D concentrations below 100 nmol/L [27], [28]. We calculated the prevalence of hypovitaminosis D under the cut-off of 75 nmol/L in our study and classified a concentration of 25(OH)D between 50–74.9 nmol/L as marginal vitamin D status (VDM).

For calcium, hypocalcaemia was defined by concentrations of ionized calcium between [1.15-0.9 mmol/L] for mild deficiency, between [0.9-0.8 mmol/L] for moderate deficiency and less than 0.8 mmol/L [29] for severe deficiency. The normal range of ionized calcium concentrations was set between 1.15 and 1.25 mmol/L and a concentration >1.25 mmol was considered as hypercalcaemia [29].

### Food Consumption Analysis

Food intakes of children and women included in this study were extracted from the food consumption data at the household level and the individual consumption of children aged less than 5 years old measured during the 2009 Food Consumption Survey, using the 24-hour recall method combined with controlled food weighing [30].

To estimate the individual consumption of women of reproductive age (19–50 years old), the energy requirement of an 18–60 year male was set as the reference (weight = 1) and used to weigh the specific energy requirements of other age and gender groups. For women 15 to 17 years of age, a weight of 1.1 was assigned, 1.4 for a pregnant or lactating woman and a weight of 0.9 for women aged 18–60 years old was used during the analyses of the 2000 and 2009 food consumption survey [31]. Different weights were also assigned to older women, adolescents and children (above 5 years of age). Household adult equivalent units were obtained by adding together all the individual adult equivalent units in the household.

First, the 24-hour recall of amount of foods consumed by the household was conducted by teams of dietitians or trained personnel from the provincial medical centers. The dietitians interviewed the women in charge of cooking the family meals. Each woman was asked to describe household food consumption in chronological order from the time the first family member of the household woke up the previous day until the same time on the day of recall. To estimate the quantities of the ingredients used for preparation of the dishes as well as quantities of consumed foods and uneaten foods, various techniques were used: 1) reproduction of the quantity of food and weighing: the mother was asked to reproduce the quantity (e.g. quantity of rice cooked using the family stock) or the volume of a food (e.g. volume of fish sauce using water). The enumerator weighed these quantities using a small digital balance; 2) use of photographic food catalogue including different serving size of typical dishes and calibrated cooking tools (spoon of vegetable oil, spoon of sugar, etc.); 3) estimation according to the prices of food purchases: food prices were converted into weight using market data. The women in charge of preparing the meals were asked whether some family members ate individual meals or snacks at home or could have eaten outside the home, and if this was the case, these members were interviewed directly and the amounts of such foods included in the 24 hour recall. The dietitian then asked which members and guests took part in each of the meals.

Secondly, for children under 5 years of age, a specific questionnaire was implemented to estimate, through the 24-hours recall, the child consumption. The dietitians interviewed the women in charge of cooking and feeding the child meals. For consumption pattern, to calculate dietary micronutrients intakes, the database of consumed food items was linked to food composition data based on the Vietnamese food composition database [32].

### Ethical Issues

The Scientific and Ethical Committees of the National Institute of Nutrition (NIN) (Hanoi, Vietnam) and of the Ministry of Health (Hanoi, Vietnam) reviewed and approved the study protocol. Before enrollment, all women were informed verbally and in writing about the aims and procedures of the study, and written informed consent was obtained from all women and children, via their mothers or guardian approval for the latter. All potential participants who declined to participate in the study were not disadvantaged in any way.

### Socioeconomic Status

Socio-economic status was calculated using the Demographic Health Statistic (DHS) Wealth Index [33], to divide households surveyed into five socio-economic quintiles: the “extreme poor” (category 1), the “poor” (category 2), the “intermediate” categories 3 and 4 and the “wealthiest” (category 5). The Wealth Index was constructed from recorded data on household assets such as tables, chairs, refrigerator, air conditioners and beds and also from housing conditions (materials of house floor, house roof, main wall) and facilities (energy for cooking, electricity and latrines), income and expenditure were not used [34].

### Data and Statistical Analysis

For biological and anthropometric status, data entry, including quality checks was performed with Excel 2007™. For consumption, the data was performed using SPSS software version 19™. Data management, including quality checks, and analysis was performed with SAS software version 9.2™ (SAS, V9.2; SAS institute, Cary, NC) [35]. Anthropometric data were entered through EpiData (version 6.0, CDC). All analyses took into account characteristics of the cluster sampling design using the appropriate survey procedures of SAS (survey freq and survey means procedures as introduced in SAS Institute Inc. 2008) [35]. Qualitative variables were expressed as percentages and standard error percentage. Continuous variables were expressed as arithmetic means and standard error of the mean except plasma vitamin D concentration, vitamin D and calcium intakes whose data were not normally distributed. Plasma vitamin D concentration and vitamin D and calcium intakes were log-transformed before statistical analysis, and are expressed as geometric means. Concentrations, prevalence of vitamin D and calcium deficiency or insufficiency and vitamin D and calcium daily intakes were estimated for each age group for children, strata, anthropometric status and socioeconomic groups for children and women.

Associations between deficiencies and relevant factors were assessed by prevalence Odds Ratios as recommended by Pearce [36]. Thus logistic regression models (surveylogistic procedure) with prevalence as the response variable were used to assess the effect of the different factors. Associations between continuous variables and relevant factors were assessed using linear regression models (surveyreg procedure). Univariate models were used to estimate crude ORs and differences. Multivariate models including relevant confounders were used to estimate adjusted ORs and differences. The first type error rate was set at 0.05.

## Results

In total, 592 women and 525 children from 592 households were included, with 50% living in an urban setting (Table 1). Anthropomorphic measurements of children living in rural areas was worse compared to children in urban areas, with lower weight, height, WAZ, WHZ, HAZ and BMI-Z-scores (P<0.05 for all, Table 1). Overall stunting prevalence in children was 23.5% and significantly greater in rural children (27.1%) than in urban children (19.1%, p<0.05). Wasting was found in 6.4% of children with no significant differences between urban (4.9%) and rural areas (7.5%; p = 0.17). Underweight prevalence was 18.5% with respectively 15.4% in urban area and 21.0% (p = 0.07) in rural area. Overweight/obesity prevalence among children was 9.5%. Among women, 19.6% were underweight, 21.5% overweight and 2.4% obese. Rural women were on average 2 years younger with a significantly lower weight and BMI than urban women but prevalence of underweight did not differ significantly between areas. In contrast, the prevalence of overweight was significantly higher in urban women (28.9%) compared to rural women (18.9%, p<0.01).

**Table 1:** Nutritional characteristics** of women and young children during the 2010 MNS.

*variable*	n***	Total	n	urban	n	rural	p value
***Women***							
Age, year	583	32.85 (0.40)	292	33.95 (0.56)	291	31.75 (9.73)	0.006
Weight, kg	583	49.1 (0.3)	292	50.2 (0.5)	291	48.0 (6.9)	0.001
Height, cm	583	152.9 (0.2)	292	153.3 (0.3)	291	152.6 (5.3)	0.123
Body mass index, kg/m^2^	583	21.0 (0.1)	292	21.3 (0.2)	291	20.6 (2.7)	0.002
***Children***							
Age, month	507	45.32 (0.90)	225	46.10 (1.15)	280	44.7 (1.14)	0.429
Weight, kg	505	14.0 (0.2)	224	14.6 (0.3)	281	13.5 (0.2)	0.001
Height, cm	506	95.8 (0.5)	225	96.9 (0.8)	281	94.9 (0.7)	0.05
WHZ*	502	−0.48 (0.05)	222	−0.29 (0.09)	280	−0.63 (0.06)	0.001
WAZ*	502	−1.01 (0.06)	221	−0.76 (0.09)	281	−1.20 (0.06)	0.000
HAZ*	497	−1.16 (0.06)	220	−0.93 (0.09)	277	−1.34 (0.07)	0.000
BMI-Z*	502	−0.36 (0.06)	221	−0.20 (0.09)	281	−0.49 (0.07)	0.009

* Body Mass Index-z-scores (BMI-z), weight-for-age (WAZ) for underweight, height-for-age (HAZ) for stunting and weight-for-height (WHZ) for wasting.

** Data are shown in mean and standard error of the mean (in parentheses).

*** Over 525 children with calcium and vitamin D data: 16 sex, 4 socioeconomic data, 8 ages, 20 weights and 19 heights are missing/over 592 women with calcium and vitamin D data: 7 socioeconomic data, 11 ages, 9 heights and 9 weights are missing.

The prevalence of poor vitamin D status of the women was very high, with the prevalence of VDD, VDI and VDM being 17%, 40% and 34%, respectively (Table 2). There was no significant difference in prevalence of vitamin D deficiency or insufficiency according to IOM standard [26] between urban and rural populations (VDD: 19.4% in urban vs 15.3% in rural, p = 0.34; VDD: 39.9% in urban vs 40.9% in rural, p = 0.83). This was still the case after the adjustment for socioeconomic group, age group and latitude. In women, vitamin D concentrations were significantly higher in overweight/obese women compared to underweight women, the concentrations in normal women being intermediate (Table 2). Consequently, the prevalence of VDD was significantly higher in underweight women compared to overweight/obese women and intermediate in normal women (p<0.01). Women in the overweight/obese category had 2.5 times lower risk (adjusted OR = 0.39, [0.20–0.78]) for VDD than the normal category with no additional risk being observed between underweight women and normal category.

**Table 2:** Calcium and vitamin D status and prevalence of deficiencies among women of reproductive age according to their anthropometric status (BMI-z)*.

		Mean Value		Prevalence					
		Calcium(mmol/L)	Vitamin D(nmol/L)	Moderate hypocalcaemia (%)	Mildhypocalcaemia (%)	Mild and Moderate hypocalcaemia (%)	VDM(%)	VDI (%)	VDD (%)
**All**	n	541	542						
	Mean/Prevalence	0.97	44.5	14.05	83.36	97.41	34.32	40.41	17.34
	IC	[0.97–0.98]	[42.31–46.80]	[10.83–17.27]	[79.89–86.84]	[96,00–98,80]	[29.74–38.90]	[35.98–44.83]	[13.16–21.53]
**Underweight**	n	104	107						
	Mean/Prevalence	0.97	41.44	16.35	82.69	99.04	30.84	38.32	24.3
	IC (me an/prevalence)	[0,95–0,98]	[38,01–45,18]	[8,69–24,00]	[74,99–90,39]	[97,13–100,00]	[22,44–39,24]	[29,08–47,56]	[15,88–32,72]
	Diff./OR	−0.01	−8.03	1.20	1.00	3.47	0.87	0.91	1.42
	Adjust. diff./OR Adjust.	−0.01	−2.40	1.14	1.08	4.26	0.80	1.02	1.32
**Normal**	n	307	299						
	Mean/Prevalence	0.97	43.73	14.01	82.74	96.74	33.78	40.47	18.39
	IC (mean/prevalence)	[0,96–0,98]	[41], [35]–[46], [26]	[9,96–18,05]	[78,29–87,18]	[94,62–98,87]	[27,75–39,81]	[34,25–46,69]	[13,35–23,44]
	Diff./OR	0	0	1	1	1	1	1	1
	Adjust. diff./OR Adjust.	0	0	1	1	1	1	1	1
**Overweight/obese**	n	124	127						
	Mean/Prevalence	0.97	49.47	11.29	86.29	97.58	38.58	40.94	9.45
	IC (mean/prevalence)	[0,96–0,99]	[45,88–53,34]	[4,57–18,01]	[78,86–93,72]	[94,77–100,00]	[29,37–47,79]	[31,21–50,68]	[3,79–15,11]
	Diff./OR	0	5.74	0.78	1.31	1.36	1.23	1.02	0.46
	Adjust. diff./OR Adjust.	0	7.38	0.72	1.39	1.23	1.30	0.97	0.39
*P*		0.59	0.001	0.6	0.73	0.48	0.43	0.9	0.01
*p Adjust.*		0.55	0.001	0.58	0.69	0.36	0.33	0.98	0.005

legend: IC: interval of confidence; p: statistical value; vitamin D means are geometric value; OR: Odds Ration; Adjust.: Adjusted.

* note: For women, underweight (BMI <18.5 kg/m2), normal (BMI 18.5–22.9 kg/m2), overweight/obesity (BMI≥23.0 kg/m2). For women: ORs were adjusted for age group, socioeconomic group, residence (urban vs rural) for calcium data and for age group, socioeconomic group, residence (urban vs rural) and latitude for vitamin D data.

Among children (Table 3), poor vitamin D status was also highly prevalent with 21% VDD, 37% VDI and 32% VDM, respectively. Prevalence of VDD, VDI and VDM did not differ significantly with the anthropometric status i.e. between stunted and non-stunted children and between underweight, normal and overweight/obese children.

**Table 3:** Calcium and vitamin D status and prevalence of deficiencies among young children according to their anthropometric status (BMI-z)*.

		Mean Value		Prevalence					
		Calcium(mmol/L)	Vitamin D(nmol/L)	Moderate hypocalcaemia (%)	Mild hypocalcaemia (%)	Mild and Moderate hypocalcaemia(%)	VDM(%)	VDI (%)	VDD (%)
**All**	n	489	485						
	Mean/Prevalence	0.99	43.36	1.23	97.55	98.77	31.55	36.7	20.62
	IC	[0.98–1.00]	[40.76–46.12]	[0.10–2.36]	[96.01–99.08]	[97,66–99,89]	[26.28–36.81]	[31.55–41.86]	[15.68–25.56]
**Underweight**	n	27	27	27	27	27	27	27	27
	Mean/Prevalence	0.99	47.68	0	96.3	96.3	44.44	25.93	18.52
	IC (mean/prevalence)	[0,96–1,02]	[37,81–60,11]	–	[88,50–100,00]	[88,50–100,00]	[23,50–65,38]	[8,92–42,93]	[2,25–34,78]
	Diff./OR	0	4.68	–	0.70	0.31	1.79	0.59	0.86
	Adjust. diff./OR Adjust.	−0.01	4.31	–	0.57	0.24	1.61	0.65	0.84
**Normal**	n	420	415						
	Mean/Prevalence	0.99	42.99	1.43	97.38	98.81	30.84	37.35	20.96
	IC (mean/prevalence)	[0,98–1,00]	[40,35–45,80]	[0,12–2,74]	[95,66–99,10]	[97,59–100,00]	[25], [32]–[36,36]	[32], [17]–[42], [53]	[15,95–25,98]
	Diff./OR	0	0	1	1	1	1	1	1
	Adjust. diff./OR Adjust.	0	0	1	1	1	1	1	1
**Overweight/obese**	n	22	20						
	Mean/Prevalence	1.01	48.78	0	100	100	35	35	15
	IC (mean/prevalence)	[0,99–1,02]	[40,21–59,18]	–	–	–	[10,23–59,77]	[4,44–65,56]	[0,00–32,60]
	Diff./OR	0.02	5.79	–	–	–	1.21	0.9	0.67
	Adjust. diff./OR Adjust.	0.01	7	–	–	–	1.30	0.80	0.61
p		0.1	0.27	<,0001	<,0001	<,0001	0.31	0.41	0.76
p Adjust.		0.41	0.21	<,0001	<,0001	<,0001	0.44	0.54	0.71

legend: IC: interval of confidence; p: statistical value; vitamin D means are geometric value; OR: Odds Ration; Adjust.: Adjusted.

* note: for children, overweight/obesity is defined as a Body Mass Index-z-scores for age (BMI-z)>2 z-scores, underweight (BMI-z)≤2 z-scores. For children: ORs were adjusted for age group, sex, socioeconomic group, residence (urban vs rural) for calcium data and for age group, sex, socioeconomic group, residence (urban vs rural) and latitude for vitamin D data.

No participant (Table 2 and 3) had severe calcium deficiency whereas prevalence of moderate hypocalcaemia affected 14% of the women and less than 1.5% of the children. In contrast, mild hypocalcaemia was found in more than 83% of women and 97% of children with only 2.6% of women and 1.2% of children being free from hypocalcaemia. In women, moderate hypocalcaemia was significantly higher in the lower socioeconomic category (28%, p<0.0001) compared to other categories (ranging from 10.7 to 15.9%).

Among women, the geometric mean consumption of vitamin D and calcium was respectively 0.15 µgday and 428.6 mg/day corresponding to about 1% of the IOM recommended nutrient intakes (RNI) for vitamin D and 42.9% of the RNI for calcium (Table 4) [26]. Mean intakes of vitamin D and calcium were higher in urban areas compared to rural areas (p<0.001 for vitamin D and p<0.01 for calcium) and increased significantly with socioeconomic categories (p<0.001 for both vitamin D and calcium). In children, the mean intake was 0.1 µg/capita/day for vitamin D and 156.5 mg/capita/day for calcium corresponding less than 1% of the vitamin D RNI and 26–31% of the calcium RNI depending on the age group [26]. Intakes increased significantly with the socioeconomic status (p<0.01 for both calcium and vitamin D). Among the potential rich calcium and/or vitamin D food groups, fatty fish and tofu were consumed by less than 23% of the women and 26% of the children while milk and milk products were consumed daily by 33.7% of the women and 20.8% of the children.

**Table 4:** Calcium and vitamin D intakes of women and children by rural and urban residence, sex, age groups and socioeconomic groups (mean±SEM and median).

	Vitamin D*			calcium *		
	n	Mean (µg)	IC	n	Mean (mg)	IC
**Women of reproductive age**						
All	579	0.15	[0,11–0,19]	579	428.63	[402,49–456,46]
**Socioeconomicstatus**						
Level 1	77	0.02	[0,00–0,05]	77	331.59	[274,55–400,47]
Level 2	88	0.05	[0,02–0,09]	88	410.57	[336,60–500,80]
Level 3	106	0.07	[0,04–0,12]	106	396.01	[324,92–482,65]
Level 4	118	0.18	[0,10–0,29]	118	416.57	[348,81–497,50]
Level 5	189	0.36	[0,24–0,51]	189	515.69	[429,13–619,64]
*p*		<0,0001			<0,0001	
**Area**						
Rural	286	0.09	[0,04–0,16]	286	396.85	[323,79–486,33]
Urban	293	0.21	[0,15–0,30]	293	462.10	[383,35–557,09]
*p*		<0.01			<0.01	
***Children***						
All	390	0.11	[0,07–0,15]	390	156.51	[136,82–179,03]
**Socioeconomicstatus**						
Level 1	99	0.04	[0,01–0,08]	99	112.07	[87,54–143,47]
Level 2	66	0.20	[0,09–0,39]	66	178.38	[137,47–231,45]
Level 3	54	0.12	[0,05–0,23]	54	152.09	[110,76–208,81]
Level 4	66	0.11	[0,04–0,22]	66	184.35	[128,5–264,44]
Level 5	105	0.12	[0,06–0,22]	105	180.85	[144,39–226,5]
*p*		<0.01			<0.01	
**Area**						
Rural	211	0.09	[0,05–0,14]	211	142.79	[107,55–189,57]
Urban	179	0.13	[0,07–0,21]	179	174.38	[139,73–217,64]
*p*		0.25			0.08	
**Sex**						
Male	203	0.11	[0,06–0,18]	203	154.42	[120,31–198,18]
Female	187	0.11	[0,05–0,17]	187	158.80	[124,79–202,11]
*p*		0.90			0.74	
**Age group**						
<6 Months	18	0.17	[0,03–0,49]	18	80.36	[32,85–196,41]
[6–12[Months	37	0.08	[0,02–0,17]	37	129.12	[89,23–186,82]
[12–24[Months	93	0.16	[0,08–0,28]	93	154.80	[112,48–213,05]
[24–36[Months	165	0.10	[0,06–0,16]	165	169.36	[131,50–218,10]
[36–60[Months	75	0.06	[0,03–0,10]	75	170.74	[129,55–225,01]
*p*		0.19			0.33	

Legend: SEP: standard of error of the prevalence; SEM: standard error of the mean; IC: interval of confidence; p: statistical value adjusted with energy intake;

* geometric value.

## Discussion

This study shows that vitamin D insufficiency (VDI) and vitamin D deficiency (VDD) and mild hypocalcaemia are a major public health concern among women in the reproductive age and young children in Vietnam. Using cut-offs recommended by IOM [26], approximately 60% of the women were vitamin D deficient or insufficient. Using the more liberal cut-off of 75 nmol/L as in many studies, 93% of the women were classified as having hypovitaminosis D. This prevalence of hypovitaminosis D is much higher in comparison to two recent studies conducted in Vietnam. A study carried out in non-pregnant women (15–49 years) living in Hanoi City and rural Hai Duong Province in northern Vietnam, indicated a mean 25(OH)D concentration of 81 nmol/L [19], much higher than the mean of 47 nmol/L in urban women in our study. The other study carried out in women aged 18–87 years sampled from various districts in Ho Chi Minh City found a mean 25(OH)D of 75.3 nmol/L [18], with 3% and 46% of the women having 25(OH)D concentrations <50 nmol/L and <75 nmol/L, respectively. The differences between these studies and our study may have several reasons. First of all, circulating 25(OH)D was measured by HPLC in the present study, considered to give reliable quantification of 25(OH)D [25] whereas the other two studies used RIA and CLIA methodology, respectively to measure 25(OH)D [37]. The difference could also be due to sampling as our study included urban women from 19 provinces all over Vietnam with different climates, food and clothing habits, and occupations and is therefore more representative of the diversity of situations in Vietnam. In our study, even if VDD prevalence was not significantly different across regions (the sample size was not calculated to allow regional comparisons). Southern regions tended to have less VDD prevalence than the others (15.7% in South regions vs 18.6% in the Central regions). In addition, the Mekong River Delta region, which includes Ho Chi Minh City, had a prevalence of VDD of 7%, in line with the prevalence of 3% reported by the study carried out only in Ho Chi Minh City [18].

Moreover, our results are in agreement with several other studies from Asia. A cross sectional study among healthy Japanese women, aged 19–66 y, working in nursing homes in the city of Niigata shows a prevalence of VDD (25(OH)D<30 nmol/L) of 42.1% in the women younger than 30 years and 10.3% in the oldest ones [38]. The mean serum 25(OH)D concentration was 34.0 nmol/L in the youngest women and 50.0 nmol/L in older ones. In another study carried out on a convenience sample of non-pregnant women aged 18–40 years living in Jakarta and Kuala Lumpur, the mean 25 (OH)D concentration was 48 nmol/L, similar to our study and over 60% of women had a concentration of vitamin D <50 nmol/L [14]. A clinical trial, in China, not regionally representative sample, shows that 40% of women from Beijing and 18% from Hong Kong have a 25(OH)D concentration <25 nmol/L and over 90% of women <50 nmol/L [13].

Similar to the women, the prevalence of vitamin D deficiency and insufficiency was high in children, with more than 50% having a 25(OH)D concentration <50 nmol/L. And 89% of the children had a 25(OH)D concentration <75 nmol/L while only 1% had a concentration >125 nmol/L. To our knowledge, this study is the first reporting data on vitamin D status in children in Vietnam. Moreover, very few data on vitamin D deficiency in children in Asia have been reported. One study carried out among infants aged 12–24 months in Shanxi province in China indicated a prevalence of VDD of 75% in spring and 8% in fall (<50 nmol/L) [39].

In general, 80–90% of vitamin D is acquired through skin synthesis under the action of sunlight and only 10–20% through diets [40]. In this study sun exposition was not measured but as in many Asian countries, Vietnamese women take radical measures to avoid sunlight exposure such as long gloves, face masks and avoid exposure between 12–14 o’clock [41]. Also, lifestyle changes (reduced outdoor work, increased covered transportation) have led to reduced sunlight exposure generally. These practices may explain in part the results, but vitamin D intakes were very low and have likely contributed to the high prevalence of hypovitaminosis D found in the present study. Indeed, the daily intakes of vitamin D of 0.15 µg/day in women fell well below the WHO RNI of 5 µg/day for adults aged 19–50 years [42] and very far below the recently revised IOM recommendation of 15 µg/day [26]. The dietary consumption data showed that only a small proportion of the population consumes vitamin D rich foods such as fatty fish which is daily eaten by <11% of women. Compared to other Asian countries, the mean intake of vitamin D in women was lower than among women from Taiwan (3.71 µg/day) [43], Hong-Kong (3.4 µg/day) [13]or Beijing, China (0.9 µg/day) [13].

In our study, 23.9% of women were overweight or obese according to criteria adapted for Asian populations (BMI≥23 kg/m^2^) and more than half of those women had a 25(OH)D concentration <50 nmol/L. This would be not surprising as different authors report that overweight/obese people commonly have a poorer vitamin D status than those with less body fat possibly due to the deposition of this vitamin in body fat compartments [44]–[46]. However, in our study the prevalence of VDD was significantly lower in overweight/obese women compared to underweight women and intermediate in women with a normal BMI. The lower severity of obesity and the higher prevalence of VDD in our study may account for this apparent discrepancy with other studies. In our study only 2.4% of women were obese (BMI >27.5 kg/m^2^) and the mean BMI of the overweight/obese category was 24.9 kg/m^2^ (IC [24.6–25.3]) whereas BMI was 28.6 kg/m^2^ in overweight/obese women in the study carried out in Spain [46] and 37 kg/m2 in obese women in the USA study [45]. Moreover, the mean 25(OH)D concentration was 43.7 nmol/L ([41.4–46.3]) in Vietnamese women with a normal BMI (mean 20.6 kg/m^2^) when it was much higher in the other two studies for similar or higher BMI. Indeed, the 25(OH)D concentration was 84.8 nmol/L in the US control subjects with a mean BMI of 21.4 kg/m^2^ [45] and 130.2 nmol/L in Spanish women with a mean BMI of 26.0 kg/m^2^ [46] highlighting the degree of low vitamin D status in our subjects.

More than 95% of the children and women had mild or moderate hypocalcaemia. To our knowledge no data on calcium deficiency is available from the South East Asian region. Calcium deficiency is primarily caused by an inadequate intake and/or poor absorption of calcium [47]. The daily consumption of calcium measured in our study was low, at less than half of recommended intake RNI [42]. The low consumption of dairy products is one of the main reasons for the low calcium intake reported here. This result is in line with studies from China [48] and Taipei [49] where women were respectively consuming less than 500 mg and 400 mg of calcium/day through diets similar to the Vietnamese diet [50] which in addition contains phytate which is known to reduce the bioavailability of calcium [51]. Among children, the consumption of rich calcium food groups was also low, with products such as milk and milk product, and tofu eaten by respectively 21% and 12.3% of the children only.

The low level of serum 25(OH)D in our subjects may have also contributed towards lowering the absorption of calcium as vitamin D facilitates the intestinal absorption of calcium by maintaining an active calcium transport across the intestine which corresponds to 85% to 90% of calcium absorption [52]. In this study, most of women and children had a 25(OH)D concentration below 75 nmol/L and the efficiency of calcium absorption increases with 25(OH)D concentrations up to approximately 80 nmol/L and then reach a plateau [52].

### Limitation of the Study

The population analyzed for this article is a subsample of the National Food Consumption Survey sample that was also used for the Nationwide Vitamin and Mineral Deficiencies Study. However, this subsample has not biased the results, as similar findings on dietary calcium intake were found among the 7,980 households surveyed [53]. Vitamin D deficiency might be season-dependent as shown in Europe [54], and the study might not be representative of the vitamin D status during the entire year. In Europe, the 25(OH)D levels were 40% higher in summer than in winter, a reflection of the differences in solar exposition. However, the current study was conducted in summer time (July – September) that is the sunniest period of the year especially in the North Vietnam making it unlikely that the current data overestimates the prevalence of vitamin D deficiency. Finally, vitamin D status is usually assessed by measuring the serum concentration of 25(OH)D through high-pressure liquid chromatography (HPLC), atmospheric pressure chemical ionization (ACPI) and mass spectrometry (MS) [37]. However, HPLC is recognized as robust, repeatable and easy to use [25]. It provides reliable quantification for routine determinations [25].

In conclusion, this study presents the first data on hypovitaminosis D in children and on hypocalcaemia in women of reproductive age and young children in Vietnam and shows that these deficiencies are highly prevalent, with possible detrimental effects on e.g. immunity and bone health. A recent study showed a high prevalence of osteoporosis (28.6% to 43.7% depending on the criteria used for diagnosis) among Vietnamese women aged >50 years old [55]. As adequate vitamin D and calcium nutrition throughout the life-course reduces the risk of osteoporosis, the high prevalence of vitamin D and calcium deficiency reported here is a likely contributor to this [56]. Strategies to improve the vitamin D and calcium status of the Vietnamese population need to be considered urgently to address this major public health concern.
